# Prevalence and Correlates of Cyberbullying Perpetration. Findings from a German Representative Student Survey

**DOI:** 10.3390/ijerph15020274

**Published:** 2018-02-06

**Authors:** Marie Christine Bergmann, Dirk Baier

**Affiliations:** 1 Criminological Research Institute of Lower Saxony, 30161 Hannover, Germany; 2 ZHAW (Zurich University of Applied Sciences) School of Social Work, 8037 Winterthur, Switzerland; dirk.baier@zhaw.ch

**Keywords:** psychological cyberbullying perpetration, sexual cyberbullying perpetration, bullying perpetration, student survey

## Abstract

Based on a survey of 9512 ninth-grade students conducted in Lower Saxony in 2013, this paper examines the prevalence of cyberbullying perpetration and the correlates of this behavior. Binary logistic multilevel regression was used in order to analyze correlates of sexual and psychological cyberbully perpetration. In the preceding semester, 2.4% of the adolescents were perpetrators of psychological cyberbullying and 0.4% bullied someone online sexually. Low levels of empathy, frequent consumption of violent media, and being victims of aggressive online behaviors are correlated with the risk that a child will become a bully. Female adolescents are less likely than boys to engage in sexual cyberbullying perpetration, but they are more likely to engage in psychological cyberbullying perpetration. Only a small share of adolescents engage in sexual and psychological cyberbullying perpetration. Both behaviors differ in their correlates, however being a victim of aggressive online behaviors increase the risk for perpetration of both behaviors, respectively.

## 1. Introduction

Almost all young people now have a mobile phone or other form of internet access. The high availability of modern communication technologies in adolescence seems desirable because it ensures access to knowledge, which is a central resource in today’s society. But, of course, teenagers, like all other age groups, use these communication technologies for more than mere knowledge acquisition and learning. There are other functions that these technologies enable and that make their use interesting. These include entertainment and social networking. The communication technologies also offer new opportunities for the exercise of aggression namely, cyberbullying.

Unlike conventional forms of bullying, which can include physical as well as verbal or relational forms of aggression, cyberbullying does not involve direct physical harm or theft/destruction of the victim’s belongings. Cyberbullying encompasses non-physical forms of aggression essentially designed to harm a person’s reputation or damage their self-esteem. The consequences for the victim of such harassment are no less drastic. In fact, because these experiences of antagonism are not limited to specific times, places, and social circles, they can greatly reduce the victims’ sense of well-being. In temporal terms, the degrading words or images cannot effectively be removed from the internet. Thus, they can remain in circulation for years to come. In spatial terms, cyberbullying cannot be readily left behind when one leaves the school building. It can follow the victims to every other area of their lives [1]. In social terms, it is unbounded because, in principle, everyone can participate in it. Indeed, it is not uncommon for people completely unknown to the victim to join in with the bullying. 

Although cyberbullying has long been the subject of scientific studies, there has to date been no representative sample done in Germany to identify how this behavior spreads. The existing studies are mostly selective; they were either conducted as online surveys, were limited to specific localities or regions, or were based on small scale samples. Online surveys are problematic because only a fraction of the potential respondents will participate. Those who participate might systematically differ in various aspects (including the use of communication technologies) from those who do not. In other words, the self-selection of respondents means that the results cannot be generalized. Beyond the lack of representative studies in Germany, at least, studies into those factors potentially influencing this behavior, involving different variables on the individual or on microsystems simultaneously, are also rare. This article therefore has two central concerns: firstly, to identify the prevalence of cyberbullying based on a representative survey of ninth-grade students in the federal state of Lower Saxony, Germany conducted in 2013. Secondly, to test for various potential correlates of this behavior to see if they actually are associated with an increased risk of engaging in cyberbullying perpetration. This seems important, as most research has been done in other cultural contexts. Germany differs from the U.S.—where most of the cyberbullying research has been conducted—since the entry of digital media into everyday youth life took place later than in the U.S. Furthermore, generally speaking, there is a lower level of aggression in Germany, which makes the question of what possible factors influencing cyberbullying perpetration appear important. 

The term cyberbullying is a specific variant of conventional bullying as defined by Olweus [2], who describes bullying as “an aggressive, intentional act or behavior that is carried out by a group or an individual repeatedly and over time against a victim who cannot easily defend him or herself.” For bullying in general, there are three characteristic features: first, the bully’s intention to cause harm; second, the fact that there is no defense available to the victim; and third, that the harmful behavior is repeated [3]. Further, it is stressed that bullying involves an imbalance of power [4]. In cyberbullying, however, this behavior does not occur in person, but is instead mediated via a communication medium (mobile phone, smartphone, internet). Physical violence, theft, and property damage are relevant bullying behaviors in the school context that cannot be afflicted on the victim online. Thus, cyberbullying behaviors are more similar to verbal or psychological bullying behavior in the school context. According to previous studies on behavior in chat rooms (for example [3,5]), it can be assumed that the following behaviors are more common types of cyberbullying: mocking, insults, threats, and the spreading of rumors.

According to the North Rhine-Westphalia study by Katzer et al. [5], 32.8% of adolescents experience at least one of these behaviors anywhere from every couple of months to several times a month, while 9.0% report weekly or even daily harassment. On the basis of previous research focusing on adolescents, it can also be assumed that the number of perpetrators is lower than the number of victims [6]. Sitzer et al. [3] were able to show that 14.1% of the adolescents surveyed reported being victims of cyberbullying, but only 12.6% identified themselves as being perpetrators. For adolescents, this is atypical, since, with conventional bullying, the number of self-reporting perpetrators usually exceeds or equals those who report themselves as victims [7,8]. There are at least two possible reasons for this difference: First, the internet makes it possible for a single perpetrator to be aggressive towards multiple victims at the same time. Degrading messages or statements can be readily posted on multiple homepages or chats. Second, the internet makes it easier to be harassed by people in other age groups. In schools, most aggression occurs between students in a single grade. Online, though, both younger and older offenders as well as age-group peers can more readily participate in bullying. The findings of Smith et al. [9] confirm this: only 57.2% of the victims said that they knew the perpetrators from school and 20.7% of the cyberbullies were completely unknown to the victims. 

With regard to possible gender differences, many studies have consistently shown that in “conventional bullying” the perpetrators are more likely to be males and the victims females [10]. The empirical findings with regard to cyberbullying, however, prove to be less consistent. While the study of Sourander et al. [11] confirms parallels to conventional bullying—with cyberbullying victims more likely to be female and offenders more likely to be male—a number of studies have found no significant gender differences between perpetrators and victims [3,9,12,13,14]. This suggests that cyberbullies are equally likely to be girls or boys. An often used line of argumentation, however, for why teenage girls could be more likely to engage in cyberbullying than boys could be the fact that it is an indirect form of aggression, something that girls are in general more likely to engage in than in direct aggression. “Boys engage in more direct physical bullying than females; and … girls engage in more indirect bullying, such as spreading rumors and manipulation of friendship” [15] (p. 19). With regard to gender differences, it should be noted that cyberbullying can take at least two forms. In addition to the already mentioned form of taunting, insulting, etc., there is cyberbullying behavior that is also explicitly sexual. It can be expected that boys are more likely to be the offenders in this sexual cyberbullying than girls. This can be concluded from the existing victim surveys. Two studies report that girls are much more frequently confronted with sexual harassment, offers, or requests than boys [16,17,18]. The perpetrators of this behavior are predominantly male internet users.

As far as correlates of cyberbullying beyond gender are concerned, current research shows that personality traits such as self-esteem or the ability to feel empathy are relevant. As two other studies show, low self-esteem is associated with more frequent cyberbullying activities [19,20]. This could be explained with the theory of social comparison processes [21]. People with low self-esteem strive to increase it. One possible way to do this is to degrade the self-esteem of others to compensate for their own low self-esteem. Cyberbullying, which includes psychological attacks, insults, and other affronts, serves this purpose well. Aggression research also supports the opposite assumption that high self-esteem leads to aggressive behavior [22]. For cyberbullying, however, a similar connection has yet to be proven [20]. The fact that lacking an ability to feel empathy tends to create perpetrators has been repeatedly shown in studies of the causes of aggressive behavior [23]. Those who cannot empathize with others cannot understand the psychological harm that their actions cause. It can therefore be assumed that a high degree of empathy would stop someone from becoming a cyberbully. Pfetsch et al. [24] were able to confirm in their study that cyberbullies are significantly less empathetic than bystanders. Other studies show that a lack of empathy and cyberbullying are related as described [25,26,27,28].

Further correlates can be found according to Bronfenbrenners ecological model in important micro systems like school and family [7]. Regarding school, it can be presumed that students who perform poorly in school are more likely to become cyberbullies. For students who spend a large part of their days in school and find their relevant peer group there, school performance provides a comparative dimension that is crucial for self-esteem. Poorer performance in school can be perceived as degrading and frustrating, leading to more frequent aggressive behaviors against others. Two studies confirm empirically this effect of poor school performance [14,29].

Additionally, Ybarra and Mitchell [14] also showed that the quality of children’s relationships with their parents can prevent or promote cyberbullying perpetration. Adolescents with weaker emotional attachment to their parents and less pronounced parental control are more likely to engage in cyberbullying perpetration. Wang et al. [30] also confirm a link between the lack of parental support and engagement in cyberbullying. In the analyses that follow, it is therefore assumed that a positive parent-child relationship reduces the likelihood of cyberbully perpetration. Parents who nurture such a relationship with their children find it easier to convey the norms of considerate togetherness. 

Another factor that could potentially influence the emergence of cyberbullying is media consumption. If this is the case, the more children consume violent media, the more likely they are to be cyberbullies. According to the general aggression model [31], the consumption of violent media can, among other things, result in the cognitive storage of aggressive behavioral scripts that are more likely to be activated in the event of conflict. Schiller et al. [32] were able to confirm these correlations with regard to cyberbullying. According to their findings, the use of violent video games has a higher correlation to cyberbullying than even to conventional bullying. They argue this occurs because the transfer of aggressive behavioral scripts is easier within the same medium. In other words, aggressive scripts learned in front of screens are more readily tapped when engaging in other online behaviors. With regard to media consumption, both the content and the duration of the exposure appear relevant. Longer periods of internet usage increase the risk of cyberbullying, it is claimed. This would also mean a greater number of contacts being made online that would simply increase the opportunities to behave in a negative way towards others [3,14,33,34]. The meta-analysis by Kowalski et al. [35] (p. 40) also confirms: “Individuals who spend more time on the Internet will (a) develop greater expertise with the use of technology and (b) probabilistically be more likely to become involved with cyberbullying as victim or perpetrator due to the time spent online.” One strength of the study used here to examine the assumptions described above is that it also allows the interrelationships between the victim and the offender and between conventional aggression in schools and cyberbullying. In this regard, the following additional assumptions can be considered:

Victims of aggression at school more frequently turn to cyberbullying. Smith et al. [9] report that victims of school bullying are almost twice as likely to become cyberbullies. Dooley et al., Hemphill et al., Raskauskas et al. and Zych et al. [1,36,37,38] have shown the same results in their studies. The internet offers bullying victims an opportunity to process their frustrations about their own negative experiences through aggressive behavior in a more or less anonymous form.

Victims of aggressive online behavior are more likely to turn to cyberbullying themselves. The switch from victim to bully is observed in various forms of aggressive behavior. It is expected that cyberbullying would reflect a similar pattern. Schulze-Krumbholz and Scheithauer [26] confirm this empirically, finding that 58.3% of cyberbullies are also victims of cyberbullying. Furthermore, aggressors in school engage more frequently in cyberbullying, too [3,17,38]. For example, Katzer [17] reports that 66.7% of school bullies also engage in cyberbullying. Smith et al. [9] confirm that perpetrators of school-based bullying are three times as likely to engage in online bullying. Adolescents with behavioral issues in one area are more likely to have them in other areas as well.

## 2. Materials and Methods

The assumptions made were tested based on a survey of students in the German state of Lower Saxony. The aim was to carry out a representative survey of approximately 10,000 ninth-grade students across the various schooling formats on offer in the state (excluding schools for students with special needs such as mental or physical disabilities, but not those for students with learning disabilities). For this survey 639 classes with 14,764 students were randomly selected. The random selection took place within each school format. Of the selected classes, 154 did not participate; the surveys were therefore administered to 485 classes with 9512 students, corresponding to a response rate of 64.4%. The surveys lasted 90 min each and were conducted in the students’ classrooms using paper-pencil questionnaires usually in the presence of a teacher or another adult supervisor. The survey was approved by the state education authority. The students’ parents were sent a one-page information sheet about the survey and were required to give their consent for their children to participate. At the beginning of the survey, the students were expressly reminded of the anonymity and voluntary nature of their responses. Accordingly, all students and parents gave their informed consent for inclusion before they participated in the study. The interviews were administered between 7 January and 5 May 2013. The make-up of the sample with regard to school formats proved to be a good reflection of the schooling situation across the state. In 2012–2013, 7.5% of all ninth-graders in Lower Saxony were enrolled in Hauptschulen (the most basic form of secondary education that usually ends with the student taking on an apprenticeship in the trades). This relates to 7.5% of the respondents to the survey, which were enrolled in Hauptschulen. The largest relative deviation was for students in special education, where there were 1.2 times more students in the general population than sampled (3.2% compared to 2.6%). The second largest deviation can be observed for Gesamtschulen (comprehensive schools) (14.4 compared to 12.7%). In order to compensate for these differences and enable representative findings for ninth graders in Lower Saxony, we opted to use data weighting. All of the prevalence results are therefore based on the weighted sample.

The sample characteristics are:

- The average age of the respondents was 14.9 years.
- 50.7% of the respondents were male. 49.3% were female.
- 10.8% of the respondents were growing up in families receiving some form of public assistance.
- 30.3% of the respondents indicated that they did not live with both of their biological parents.
- 24.3% have a “migrant background”, with the two largest groups being children born in or descended from at least one migrant from the countries of the former Soviet Union countries and Turkey. The definition of migrant used in the study included those respondents not born in Germany, those who do not have German citizenship, and/or for whom this applies to at least one biological parent.

The survey addressed a multitude of topics and was not dedicated exclusively to cyberbullying perpetration and its correlates. This means that, in some cases, only short instruments were used to record data about the different constructs. In addition, in schools for students with learning disabilities, the entire range of topics could not be queried, because the survey would have taken too long (and the students would not have remained sufficiently focused to provide accurate responses). Therefore, a shortened survey form was administered to these students. This shorter form did not inquire about respondents’ own cyberbullying activities, but only if they had been the victims of cyberbullying. Consequently, the following analyses about those who engage in cyberbullying do not include these students. Finally, it should be pointed out that this is a cross-sectional survey that ultimately does not allow conclusions to be drawn about cause-and-effect relationships. 

The central variable of the investigation was engagement in acts of cyberbullying. Based on Sitzer et al. [3], six statements were included in the survey that were to be answered with regard to the most recent semester. Students were asked to identify the frequency of such occurrences on a scale of “1—never” to “6—several times a week”. They were asked how often they had

1. ridiculed, insulted, abused, or threatened others online;
2. spread rumors about other people or dissed them online;
3. posted others’ private messages, confidential information, photos, or videos online in order to out them or ridicule them;
4. excluded others from an online group;
5. sent unwanted photos or videos of nude persons or had engaged in unwanted discourse about sex with others;
6. encouraged others to engage in sexual (or sexualized) acts against their will, such as undressing in front of a webcam.

Using Mplus 7.4 (Muthen and Muthen, Los Angeles, CA, USA), two confirmatory factor analyses including the six cyberbullying items were conducted. In the first analysis all six items were forced to load on one factor; the fit indices of this model were not satisfactory (Chi2 = 267.926, df = 8, CFI = 0.817, TLI = 0.657, RMSEA = 0.060, SRMR = 0.072). In the second model the items loaded on two correlated factors: the first four items loaded on the factor “psychological cyberbullying”, the last two items on the factor “sexual cyberbullying”. The fit indices of this two factor model were significantly better (Chi2 = 35.267, df = 7, CFI = 0.980, TLI = 0.957, RMSEA = 0.021, SRMR = 0.024). Factor loadings varied between λ = 0.53 and λ = 0.78 (psychological cyberbullying) and λ = 0.68 and λ = 0.86 (sexual cyberbullying); the correlation between both factors was φ = 0.63. Because of the results of the confirmatory factor analyses, the two forms of cyberbullying are analyzed separately in the following.

The various correlates were operationalized as follows. To assess adolescents’ self-esteem, a subscale of the KINDL [39,40] questionnaire was used. The scale includes four items (e.g., “I was proud of myself last week.”) to be rated on a five-point scale (see Table 1). The mean of the responses was 3.19, which is slightly above the theoretical mean. The majority of the adolescents thus report a higher level of self-esteem. The reliability of the scale is satisfactory considering the small number of items. The teenagers’ empathy was also measured with four items (e.g., “I am very moved when I see someone crying”). These are based on an inventory for measuring impulsivity, risk-taking, and empathy designed for children aged 9 to 14 [41]. The reliability of the scale was good, with a Cronbach’s alpha of 0.80; the mean response was 2.94. In order to record the respondents’ performance in school, they were asked about the last report card grade they received in the subjects of mathematics, German, history, and biology (on the usual German scale where 1 is excellent and 6 is failing). The mean for all four subjects was 3.03 (Cronbach’s alpha = 0.73). To obtain information about the parent-child relationship, a scale was used to measure parental care. Respondents were asked with regard to their pre-teen years to indicate how often their mother or father consoled them when they were sad. The six items [42] (p. 119) were answered on a five-point Likert scale. These items were queried separately for the mother and father. A mean value for each item was then calculated for both parents and then the mean over all six items. The reliability of the scale was good. The overall mean of 4.00 indicates that most adolescents received a high level of attention. With regard to violent media consumption, five items queried the consumption of violent movies (horror films restricted to ages 16+, those restricted to ages 18+, and other films restricted to 18+) as well as violent games (first- and third-person shooter games, fighting games). The respondents were given a range of answers from “1—never” to “7—daily,” with a mean of 2.55. The reliability of the scale was good. The internet usage of respondents was queried using an open-ended question. The respondents were asked to indicate how long (in hours and minutes) they used social networks or chatted on both a typical school day and a typical weekend day. The responses for the former being multiplied by five and those for the latter doubled, added together and divided by seven to obtain a figure for average daily usage. On average, the respondents spend three hours and 38 min a day on social networks and chatting.

In addition to these scales, four additional variables were included in the analyses. The following dummy variables, where students who experienced at least one of the behaviors in question were assigned the value 1, and the rest the value 0, were created:

- The dummy variable “victims of aggression in school” is based on six behaviors [8] (p. 57) (These were as follows: “I was intentionally beaten or kicked by other students.” “Other students teased me or said ugly things about me.” “Other students purposely ruined my things.” “Other students blackmailed me and forced to give away money or things.” “I was excluded from joint ventures because other students wanted me to be excluded.” “Other students treated me like air and purposely ignored me.” The question set was introduced as follows: “How many times did you experience the following things during the previous semester?”). The variables were dichotomized. If a respondent indicated he or she experienced one or more of the six behaviors, the variable was assigned a value of 1. At least 52.7% of the respondents had experienced one of these aggressions in the previous semester (Table 2).
- Whether the respondents had been victims of online aggression was queried with the same questions as to those who had engaged in such activities in the past semester [3]. A distinction was drawn between psychological and sexual bullying. Again, the corresponding variables were dichotomized. A value of 1 was assigned to the corresponding variables, if the respondent indicated they experienced at least one of the activities. Thus, 40.4% of adolescents said that they had been the target of at least one online act of psychological aggression during the semester, while 13.5% reported experiencing at least one act of sexual aggression.
- The dummy variable “perpetrator of aggression in school” is based on the same items, which asked if the respondents had experienced such aggression. The variable was created in the same way as the previously described indices. 58.9% of the respondents said that they had engaged in at least one form of aggression in school during the past semester.

## 3. Results

### 3.1. Prevalence of Cyberbullying

The proportions of respondents who indicated they had engaged in cyberbullying in the past semester are shown in Table 3. The table shows both those who said that they had only rarely engaged in such acts and those who admitted to engaging in such acts frequently. Respondents who indicated that they had committed such acts “Once or twice” or “three to six times” in the past semester were classed as rarely engaging in such activities. A defining feature of cyberbullying is its repeated occurrence. We set the frequency of aggression that constitutes bullying based on the recommendations of Solberg and Olweus [43], who suggest a threshold of two to three times a month, corresponding to the “several times a month” in this survey. Thus, cyberbullies were those who admitted to engaging in such behaviors “several times a month”, “once a week”, or “several times a week”. We label this group in Table 3 as those who engage in those behaviors frequently. According to this definition, 2.4% (*n* = 216, weighted data) of the adolescents surveyed could be defined as cyberbullies. Mocking and the spreading of rumors are the more common forms of such cyberbullying. For sexual cyberbullying, 0.4% (*n* = 38) of the respondents can be classified as perpetrators. Both forms of sexual cyberbullying occur with approximately equal frequency.

In order to examine whether the perpetrator rates differ significantly from the victim rates for online aggressive behaviors, variance analyses with measurement repetition were performed. For this purpose, the overall rates of aggressive behavior in perpetrators and victims were used. Firstly, the variance analyses show that the proportion of perpetrators is significantly lower than the proportion of victims (26.7 to 40.5%, F = 701,636, *p* < 0.001). Secondly, with respect to sexually aggressive behavior, the proportion of offenders is also significantly lower than the proportion of victims (2.4 to 13.5%, F = 950,499, *p* < 0.001). With regard to the gender differences among cyberbullies, 2.9% of the male respondents indicated that they engaged in psychological cyberbullying, while only 1.9% of the girls are perpetrators of psychological cyberbullying. This difference is significant (Cramer’s V = 0.032, *p* < 0.01). There is also a significant gender difference with regard to sexual cyberbully perpetration: 0.6% of the boys admitted to engaging in this behavior at least several times a month, while only 0.2% of the female respondents reported to have committed it (Cramer’s V = 0.034, *p* < 0.01).

### 3.2. Correlates of Cyberbullying Perpetration

In order to investigate the links between potential correlates and the prevalence of cyberbullying, two multivariate analyses were carried out. Since the dependent variables are binary (engaged in such behavior at least several times per month: no vs. yes), logistic regression analyses were run. In addition, because the survey was conducted within specific school classes, the clustering of pupils within these classes has to be considered when analysing the data. This is made possible by using multilevel analyses. To avoid a high number of missing cases when deleting cases listwise, a multiple imputation strategy was applied. Using Mplus 7.4 missing values were imputed via Bayesian estimation. This was done 20 times, meaning that 20 new datasets with imputed values were calculated (for categorical variables integer values were imputed). For the analyses parameter estimates were averaged over the 20 sets of analyses. Table 4 presents the results of the binary logistic multilevel regression analyses. The analyses include 9512 pupils in 485 classes. In addition to the conventional significance levels, the 10% level is also reported. Due to the sample size, this may seem unusual. It should be noted, however, that only very few respondents (*n* = 38) reported engaging in sexual cyberbullying. This makes the identification of significant effects more difficult.

The type of school did not prove to be a significant correlate. In other words, both forms of cyberbullying were more or less equally prevalent across all types of schools. This is particular to cyberbullying, as there are usually significant differences with regard to school-based bullying, where higher rates of engaging in such behaviors are found in lower-level forms of school [8] (p. 88). 

On the one hand, looking at gender, female respondents engage in sexual cyberbullying significantly less frequently than males. On the other hand, and in contradiction to the bivariate analyses, female adolescents are significantly more likely to engage in psychological cyberbullying. In the multivariate model girls have a greater risk than boys of becoming perpetrators of psychological cyberbullying because factors such as empathy have been controlled. Girls are generally more empathetic than boys; their level of aggression is therefore generally lower than that of boys, if the ability to feel empathy and cyberbullying are correlated. If the genders are compared while controlling for such third factors, there is a higher load on the girls, which is consistent with findings that girls are more prone to indirect forms of aggression and would rather avoid direct, physical confrontation. Self-esteem is slightly associated with an increased risk of psychological cyberbully perpetration; it does not correlate to sexual cyberbullying. A higher level of empathy is significantly associated with a reduced risk for both forms of cyberbully perpetration. Poor school grades are associated with more frequent cyberbully perpetration, but the effect is only significant with regard to psychological bullying. The study also confirmed that parental attention is negatively associated with cyberbullying, at least for psychological forms of cyberbully perpetration. A more frequent consumption of violent media and generally longer time spent online are correlated to a higher frequency of engaging in cyberbullying. Being a victim of aggression at school does not correlate to engaging in cyberbullying. There is only a slight effect for psychological bullying where victims are less likely to engage in cyberbullying. Experiencing online aggressive behavior, however, does show significant effects, but the strength varies. For example, those who experience psychological aggression are more likely to engage in psychological cyberbullying than in sexual cyberbullying. The reverse applies to those who experience acts of sexual aggression. These are more closely related to sexual cyberbullying and less closely tied to psychological cyberbullying. Finally, it can be seen that those who engage in aggressive behaviors at school are significantly more likely to engage in cyberbullying, with an effect for psychological and sexual cyberbullying. All in all, engaging in cyberbullying can be explained quite well with these variables. The models explain 46.4% of the variance in psychological cyberbullying and 46.7% of that in sexual cyberbullying.

## 4. Discussion

The findings of a representative survey of ninth-grade students in the German state of Lower Saxony shows that online aggressive behavior is a relevant problem. In the past semester, 26.7% of the respondents engaged in at least one act of online psychological aggression and 2.4% engaged in its sexual counterpart. Furthermore, 2.4% of the respondents can be labeled psychological cyberbullies, defined as engaging in this behavior at least several times a month, while 0.4% are sexual cyberbullies. The most prevalent form of cyberbullying is mocking and spreading rumors about others. These prevalence rates are lower, compared to other studies in other cultural settings [7]. However, due to the different conceptualization and operationalization the prevalence rates of cyberbully perpetration are not easily comparable [44]. At the same time, the analyses show that aggressive behavior is even more widespread in the school setting, where 58.9% of the respondents indicated that they had engaged in such behavior in the past semester. This is in line with other studies reporting that bullying and aggression in schools are more prevalent than those behaviors carried out online [7].

In this respect, measures to prevent bullying in schools appear necessary. The findings also suggest that such measures could also reduce cyberbullying perpetration. If preventive measures target aggressive behavior by working to increase the children’s ability to feel empathy, this should also prevent cyberbullying, since a strong correlation exists between the two [45]. At the same time, there are significant relationships between aggressive behaviors in school and cyberbullying. There is a highly significant correlation between engaging in aggressive behaviors in school and engaging in psychological cyberbullying. This has been shown also by other studies [46]. Prevention programs that address aggression in school generally might also reduce the occurrence of cyberbullying.

Sexual cyberbullying perpetration, by contrast, is largely not correlated to such aggressive behaviors in school. Indeed, it is primarily those adolescents with little record of acting out in school who engage in sexual cyberbullying. The correlation between being a victim of and a perpetrator of sexual cyberbullying is particularly strong. Sexual cyberbullying is thus centered on a small group who move between being the bullies and being the victims themselves. Further studies about this small group of adolescents therefore seems desirable.

According to previous research, females are less likely to engage in sexual cyberbullying [3,16]. With regard to psychological cyberbullying perpetration, contrary to the assumption made, it appears that girls, when various background factors are controlled, are more frequently perpetrators than boys. In the end, girls are more likely to use indirect and verbal forms of aggression than direct or physical forms of violence.

With regard to empathy, the consumption of violent media, school grades, and time spent online, our results confirm that these are important predictors of cyberbullying [28,38,46]. Pupils with less empathy, more frequent use of violent media, lower grades, and more time spent online are more likely to engage in psychological or, in some cases, sexual cyberbullying perpetration.

The parent-child relationship is associated with cyberbullying perpetration, as expected. Those who receive greater parental attention are less likely to engage in psychological cyberbullying. Sexual cyberbullying perpetration, on the other hand, is not correlated to school grades or internet usage. In general, it must be noted that the parental influence could be conveyed by other factors. Parenting choices can, for example, influence factors such as empathy or an affinity to violent media. These factors are closely related to cyberbullying perpetration. Such indirect correlations can be visualized through structural equation models that are still rarely found in cyberbullying research, almost certainly due to the lack of longitudinal studies. Further investigation into the lack of correlation between self-esteem and cyberbullying perpetration is also needed.

In terms of self-esteem, it has been suggested that low self-esteem could result in seeing oneself as worse off than others, resulting in frustration and trying to compensate for this by cutting others down to size. This argument might not apply in two respects. Firstly, low self-esteem may not be the negative experience it is claimed to be (see [22]). For adolescents, it is not atypical to doubt themselves during puberty. Consequently, these perceptions do not necessarily result in a pressure to compensate. Secondly, cyberbullying may not be the preferred strategy to deal with negative self-esteem. Adolescents with low self-esteem may be less able to direct their aggression against others than against themselves. Compensation strategies would then be more likely to be in the form of alcohol and drug use, for example.

Finally, we need to address the various limitations to the validity of the analyses presented here. Since this is a cross-sectional study, the question of cause and effect cannot be answered. This question is important, especially with regard to the overlapping of victims and perpetrators. The results show that victims of online aggression are themselves engaging in cyberbullying perpetration. The causal direction could also be the other way around. Only longitudinal studies will be able to present this cycle in a differentiated way. Another limitation relates to the instruments used. As stated, the survey was not primarily about cyberbullying. This is reflected in the operationalization of the cyberbullying with only six items, which, of course, only cover a limited range of behaviors. Furthermore, the aspect of imbalance of power—which is one of the defining aspects of cyberbullying [35]—is not covered in the operationalization used in this study. In addition, only short scales could be included to measure the influencing factors. Some of the scales show rather low alpha reliabilities, for example, self-esteem or grades. This is partly due to the relatively low number of items on which the scales are based (between four or six items). Regarding self-esteem, it should be noted that we used the KINDL questionnaire [33,34], the reliability of which has been proven in the past. Finally, it should be noted that only selected conditional factors could be tested, since factors such as the aggressiveness or bullying behavior of friends—both potentially further influences on cyberbullying—were not queried.

## 5. Conclusions

The findings of a representative survey of ninth-grade students in the German state of Lower Saxony shows that online aggressive behavior is a relevant problem. Defined as engaging in this behavior at least several times a month, 2.4% of the respondents can be classified as psychological cyberbullies, while 0.4% are classified as sexual cyberbullies. The most prevalent form of cyberbullying is mocking and spreading rumors about others.

The prevalence of psychological cyberbullying perpetration is correlated with being female and having high self-esteem, low empathy, bad grades, a low level of parental care, the consumption of violent media, the experience of aggression online (psychological and sexual) and at school, and the perpetration of aggression at school. The prevalence of sexual cyberbullying is correlated with being male, having low empathy, violent media consumption, and the experience of psychological and sexual aggression online.

## Figures and Tables

**Table 1:** Descriptive statistics of the correlates (1).

Correlates	Range	Cronbachs α	Mean	Stdev.
Self-esteem	1–5	0.64	3.19	0.76
Empathy	1–4	0.80	2.94	0.68
Grades	1–6	0.73	3.03	0.69
Parental care	1–6	0.89	4.00	0.75
Violent media consumption	1–7	0.79	2.55	1.26
Internet usage (in h:min)	-	-	03:38	04:35

**Table 2:** Descriptive statistics of the correlates (2).

Factor	Number of Items	Percentage Experienced/Carried Out at Least Once in the Last Semester (%)
Victims of aggression in school	6	52.7
Victims of online aggression	4	40.5
Victims of sexual online aggression	2	13.5
Perpetrator of aggression in school	6	58.9

**Table 3:** Cyberbullying prevalence rate (in %, weighted data).

Type of Cyberbullying	Cyberbully Behavior	Rarely	Frequently	Total (Committed at Least Once)
Psychological cyberbullying	Mocked someone	17.4	1.7	19.1
	Spread rumors	12.4	1.0	13.4
	Humiliated someone	4.4	0.4	4.8
	Excluded s.o. online	5.7	0.4	6.1
	Total	24.3	2.4	26.7
Sexual cyberbullying	Sent photos/videos or talked about sex online	1.7	0.3	2.0
	prompted to engage in sexual activites	0.9	0.2	1.1
	Total	2.0	0.4	2.4

**Table 4:** Correlates of the prevalence of cyberbully perpetration (binary logistic multilevel regression, show: unstandardized coefficients).

Correlates	Psychological Cyberbullying	Sexual Cyberbullying
Pupil level		
female	0.362 ^†^	−0.998 ^†^
Self-esteem (z)	0.243 *	−0.102
Empathy (z)	−0.525 ***	−0.774 **
Grades (z)	0.502 ***	0.365
Parental care (z)	−0.268 **	0.077
Violent media consumption (z)	0.482 ***	0.312 *
Internet usage (in h:min) (z)	0.030 *	0.035
Victim of aggression in school	−0.334 *	−0.408
Victim of online aggression	1.450 ***	0.732 ^†^
Victim of sexual online aggression	0.384 *	2.623 ***
Perpetrator of aggression in school	1.726 ***	0.940 ^†^
Class level		
School type: lowest level	reference	reference
School type: medium level	0.030	−0.127
School type: highest level	0.288	0.155
N pupil	9512	9512
N classes	485	485
R^2^ (pupil level)	0.464	0.467

(z)—variables were centered at the grand mean; ^†^ *p* < 0.10; * *p* < 0.05; ** *p* < 0.01; *** *p* < 0.001.

## References

[1] Dooley J.J., Pyzalski J., Cross D. (2009). Cyberbullying versus face to face bullying. A theoretical and conceptual review. Z. Psychol. J. Psychol..

[2] Olweus D. (1993). Bullying at School. What We Know and What We Can Do.

[3] Sitzer P., Marth J., Kocik C., Müller K.N. (2012). Ergebnisbericht der Online-Studie, Cyberbullying bei Schülerinnen und Schülern.

[4] Wolke D., Lereya S.T. (2015). Long-term effects of bullying. Arch. Dis. Child..

[5] Katzer C., Fetchenhauer D., Belschak F. (2009). Cyberbullying in Internet-Chatrooms–Wer sind die Täter? Ein Vergleich von Bullying in Internet-Chatrooms mit Bullying in der Schule aus der Täterperspektive. Z. Entwicklungspsychol. Pädag. Psychol..

[6] Beran T., Mishna F., McInroy L., Shariff S. (2015). Children’s Experiences of Cyberbullying: A Canadian National Study. Nat. Assoc. Soc. Work..

[7] Modecki K.L., Minchin J., Harbaugh A.G., Guerra N.G., Runions K.C. (2014). Bullying prevalence across contexts: A meta-analysis measuring cyber and traditional bullying. J. Adolesc. Health.

[8] Baier D., Pfeiffer C., Simonson J., Rabold S. (2009). Jugendliche in Deutschland als Opfer und Täter von Gewalt. Erster Forschungsbericht zum Gemeinsamen Forschungsprojekt des Bundesministeriums des Inneren und des KFN.

[9] Smith P.K., Mahdavi J., Carvalho M., Fisher S., Russel S., Tippett N. (2008). Cyberbullying: Its nature and impact in secondary school pupils. J. Child Psychol. Psychiatry.

[10] Olweus D., Limber S.P. What we know about bullying: Information form the Olweus bullying questionaire. Proceedings of the Annual Meeting of the International Bully Prevention Assosication.

[11] Sourander A., Brunstein Klomek A., Ikonen M., Lundroos J., Luntamo T., Koskelainen M., Ristkari T., Hellenius H. (2010). Psychosocial Risk Factors Associated with Cyberbullying among Adolescents. Arch. Gen. Psychiatry.

[12] Hinduja S., Patchin J.W. (2008). Cyberbullying: An explanatory analysis of factors related to offending and victimization. Deviant Behav..

[13] Slonje R., Smith P.K. (2008). Cyberbullying: Another main type of bullying?. Scand. J. Psychol..

[14] Ybarra M., Mitchell K.J. (2004). Online Aggressor/targets, Aggressors, and Targets: A Comparison of Associated Youth Characteristics. J. Child Psychol. Psychiatry Allied Discip..

[15] Wimmer S. (2009). Views on gender differences in bullying in relation to language and gender role socialization. Griffith Working Papers in Pragmatic and Intercultural Communications.

[16] Grimm P., Thein S., Clausen-Muradian E., Koch E. (2008). Gewalt im Web. 2.0. Der Umgang Jugendlicher mit Gewalthaltigen Inhalten du Cyber-Mobbing Sowie die Rechtliche Einordnung der Problematik.

[17] Katzer C. (2007). Gefahr aus dem Netz. Der Internet-Chatroom als Neuer Tatort für Bullying und Sexuelle Viktimisierung von Kindern und Jugendlichen. Ph.D. Thesis.

[18] Wachs S., Wolf K.D., Pan C. (2012). Cybergrooming: Risk factors, coping strategies and associations with cyberbullying. Psicothema.

[19] Frisén A., Jonsson A.-K., Persson C. (2007). Adolescents’ perception of bullying: Who is the victim? Who is the bully? What can be done to stop bullying?. Adolescence.

[20] Hinduja S., Patchin J.W. (2010). Cyberbullying and self-esteem. J. Sch. Health.

[21] Frey D., Irle M. (1993). Theorien der Sozialpsychologie. Band 1.

[22] Baumeister R.F., Smart L., Boden J.M. (1996). Relation of threatenend egotism to violence and aggression: The dark side of high self-esteem. Psycol. Rev..

[23] Hosser D., Beckurts D. (2005). Empathie und Delinquenz.

[24] Pfetsch J., Müller C.R., Ittel A. (2014). Cyberbullying und Empathie: Affektive, kognitive und meidenabsierte Empathie im Kontext von Cyberbullying im Kindes- und Jugendalter. Diskurs Kindh. Jugendforsch..

[25] Steffgen G., König A., Pfetsch J., Melzer A. (2011). Are cyberbullies less empathic? Adolescents’ cyberbullying behavior and empathic responsiveness. Cyberpsychol. Behav. Soc. Netw..

[26] Schultze-Krumbholz A., Scheithauer H. (2009). Social-behavioral correlates of cyberbullying in a German student sample. Z. Psychol. J. Psychol..

[27] Ang R.P., Goh D.H. (2010). cyberbullies among adolescents: The role of affective and cognitive empathy, and gender. Child Psychiatry Hum. Dev..

[28] Del Rey R., Lazuras L., Casas J.A., Barkoukis V., Ortega-Ruiz R., Tsorbatzoudis H. (2015). Does empathy predict (cyber) bullying perpetration, and how do age, gender and nationality affect this relationship?. Learn. Individ. Differ..

[29] Kowalski R.M., Limber S.P. (2013). Psychological, physical, and academic correlates of cyberbullying and traditional bullying. J. Adolesc. Health.

[30] Wang J., Iannotti R., Nansel T.R. (2009). School Bullying among Adolescents in the United States: Physical, Verbal, Relational, and Cyber. J. Adolesc. Health.

[31] Anderson C.A., Bushman B.J. (2002). Human Aggression. Ann. Rev. Psychol..

[32] Schiller E.M., Gradinger P., Strohmeier D. (2014). Nutzung gewalthaltiger Bildschirmspiele als längsschnittlicher Risikofaktor für Cyberbulling in der frühen Adoleszenz. Diskurs Kindh. Jugendforsch..

[33] Didden R., Scholte R.H.J., Korzilius H., De Moor J.M.H., Vermeulen A., O’Reilly M., Lang R., Lancioni G.E. (2009). Cyberbullying among students with intellectual and developmental disability in special education setting. Dev. Neurorehabil..

[34] Twyman K., Saylor C., Taylor L.A., Comeaux C. (2010). Comparing children and adolescents engaged in cyberbulling to matched pairs. Cyberpsychol. Behav. Soc. Netw..

[35] Kowalski R.M., Giumetti G.W., Schroeder A.N., Lattanner M.R. (2014). Bullying in the digital age: A critical review and meta-analysis of cyberbullying research among youth. Psychol. Bull..

[36] Hemphill A., Kotevski A., Tollit M., Smith R., Herrenkohl T.I., Toumbourou J.W., Catalano R.F. (2012). Longitudinal predictors of cyber and traditional bullying perpetration in Australian secondary school students. J. Adolesc. Health.

[37] Raskauskas J., Stoltz A.D. (2007). Involvement in traditional an electronic bullying among adolescents. Dev. Psychol..

[38] Zych I., Ortega-Ruiz R., Del Rey R. (2015). Systematic review of theoretical studies on bullying and cyberbullying: Facts, knowledge, prevention, and intervention. Aggress. Viol. Behav..

[39] Ravens-Sieberer U., Bullinger M., Schuhmacher J., Klaiberg A., Brähler E. (2003). Der Kindl-R-Fragebogen zur Erfassung der gesundheitsbezogenen Lebensqualität bei Kindern und Jugendlichen—Revidierte Form. Diagnostische Verfahren zur Lebensqualität und Wohlbefinden.

[40] Ravens-Sieberer U., Ellert U., Erhart M. (2007). Gesundheitsbezogene Lebensqualität bei Kindern und Jugendlichen in Deutschland. Eine Normstichprobe für Deutschland aus dem Kinder und Jugendsurvey (KIGGS). Bundesgesunheitsbl. Gesundheitsforsch. Gesundheitsschutz.

[41] Stadler C., Janke W., Schmeck K. (2004). IVE—Inventar zur Erfassung von Impulsivität, Risikoverhalten und Empathie bei 9-bis 14-jährigen Kindern.

[42] Baier D., Pfeiffer C., Mößle T., Pfeiffer C., Baier D. (2014). Elterliches Erziehungshandeln im Geschlechtervergleich. Die Kriese der Jungen. Phänomenbeschreibung und Erklärungsansätze.

[43] Solberg M., Olweus D. (2003). Prevalence estimation of school bullying with the Olweus Bully/Victim Questionaire. Agress. Behav..

[44] Patchin J.W., Hinduja S. (2015). Measuring cyberbullying: Implications for research. Aggress. Viol. Behav..

[45] Brewer G., Kerslake J. (2015). Cyberbullying, self-esteem, empathy and loneliness. Comput. Hum. Behav..

[46] Baldry A.C., Farrington D.P., Sorrentino A. (2016). Cyberbullying in youth: A pattern of disruptive behaviour. Psicol. Educ..

